# Development of Kupffer cell targeting type-I interferon for the treatment of hepatitis via inducing anti-inflammatory and immunomodulatory actions

**DOI:** 10.1080/10717544.2018.1464083

**Published:** 2018-04-24

**Authors:** Yuki Minayoshi, Hitoshi Maeda, Hiroki Yanagisawa, Keisuke Hamasaki, Yuki Mizuta, Kento Nishida, Ryo Kinoshita, Yuki Enoki, Tadasi Imafuku, Victor Tuan Giam Chuang, Tomoaki Koga, Yukio Fujiwara, Motohiro Takeya, Kayoko Sonoda, Tomohiko Wakayama, Kazuaki Taguchi, Yu Ishima, Tatsuhiro Ishida, Yasuko Iwakiri, Motohiko Tanaka, Yutaka Sasaki, Hiroshi Watanabe, Masaki Otagiri, Toru Maruyama

**Affiliations:** a Department of Biopharmaceutics, Graduate School of Pharmaceutical Sciences, Kumamoto University, Kumamoto, Japan;; b School of Pharmacy, Monash University Malaysia, Bandar Sunway, Malaysia;; c Department of Molecular Medicine, Graduate School of Pharmaceutical Sciences, Kumamoto University, Kumamoto, Japan;; d Department of Cell Pathology, Graduate School of Medical Sciences, Kumamoto University, Kumamoto, Japan;; e Department of Histology, Graduate School of Medical Sciences, Kumamoto University, Kumamoto, Japan;; f Faculty of Pharmaceutical Sciences and DDS Research Institute, Sojo University, Kumamoto, Japan;; g Department of Pharmacokinetics and Biopharmaceutics, Institute of Biomedical Sciences, Tokushima University, Tokushima, Japan;; h Department of Internal Medicine, Sections of Digestive Diseases, Yale University School of Medicine, New Haven, CT, USA;; i Department of Gastroenterology and Hepatology, Graduate School of Medical Sciences, Kumamoto University, Kumamoto, Japan

**Keywords:** Type-I interferon, Kupffer cell, albumin fusion technology, mannose, anti-inflammation, immunomodulation

## Abstract

Because of its multifaceted anti-inflammatory and immunomodulatory effects, delivering type-I interferon to Kupffer cells has the potential to function as a novel type of therapy for the treatment of various types of hepatitis. We report herein on the preparation of a Kupffer cell targeting type-I interferon, an albumin-IFNα2b fusion protein that contains highly mannosylated N-linked oligosaccharide chains, Man-HSA(D494N)-IFNα2b, attached by combining albumin fusion technology and site-directed mutagenesis. The presence of this unique oligosaccharide permits the protein to be efficiently, rapidly and preferentially distributed to Kupffer cells. Likewise IFNα2b, Man-HSA(D494N)-IFNα2b caused a significant induction in the mRNA levels of IL-10, IL-1Ra, PD-L1 in RAW264.7 cells and mouse isolated Kupffer cells, and these inductions were largely inhibited by blocking the interferon receptor. These data indicate that Man-HSA(D494N)-IFNα2b retained the biological activities of type-I interferon. Man-HSA(D494N)-IFNα2b significantly inhibited liver injury in Concanavalin A (Con-A)-induced hepatitis model mice, and consequently improved their survival rate. Moreover, the post-administration of Man-HSA(D494N)-IFNα2b at 2 h after the Con-A challenge also exerted hepato-protective effects. In conclusion, this proof-of-concept study demonstrates the therapeutic effectiveness and utility of Kupffer cell targeting type-I interferon against hepatitis via its anti-inflammatory and immunomodulatory actions.

## 1. Introduction

Interferon is currently in widespread use in clinical settings for treating various diseases such as viral hepatitis, owing to its excellent biological activity. Interferon acts by binding to its receptors on cells, eliciting different effects, depending on the cell type. For example, anti-viral and anti-tumor effects are produced when interferon acts on parenchymal cells, but when acting on immunocompetent cells such as macrophages and T cells, it shows anti-inflammatory and immunomodulatory effects, limiting tissue damage and preventing autoimmunity (Ivashkiv & Donlin, 2014).

Inflammatory and an immune response are common in the pathogenesis of diverse forms of liver diseases that lead to liver damage, steatosis and fibrosis (Tilg et al., 2006; Albano & Vidali, 2010; Bieghs & Trautwein, 2013). It was recently reported that inflammasomes contribute to the onset and development of liver diseases and that intrahepatic macrophages cause liver inflammation through inflammasome signaling (Lohse et al., 2010; Wree et al., 2014; Tilg et al., 2016). Interestingly, under hepatopathic conditions, endogenous type-I interferon acts specifically on Kupffer cells which are macrophages residing in the liver, thus promoting the induction of IL-10, an anti-inflammatory cytokine, and IL-1Ra, an inhibitor of the IL-1 signal produced by inflammasomes, therefore functioning as an endogenous protective factor against liver damage (Ziegler-Heitbrock et al., 2003; Guarda et al., 2011; Petrasek et al., 2011; Roh et al., 2014). In fact, studies using non-viral hepatitis animal models such as alcohol-induced hepatitis and nonalcoholic steatohepatitis demonstrated that exogenous IL-10 or IL-1Ra ameliorates hepatic injury (Petrasek et al., 2012). Type-I interferon also suppresses T-cell activation by affecting PD-1/PD-L1 interaction through increasing the expression of PD-L1 in monocytes, including macrophages (Shaabani et al., 2016). Since an exaggerated T-cell activation results in the development of tissue damage, it is possible that the induction of PD-L1 by type-I interferon in Kupffer cells could ameliorate liver damage via the development of immune tolerance.

Type-I interferon possesses the potential to be used as a novel therapeutic strategy for the treatment of various types of hepatitis, due to its anti-inflammatory and immunomodulatory effects. The delivery of type-I interferon to Kupffer cells therefore would be a desirable drug targeting strategy. However, type-I interferon lacks the ability to recognize Kupffer cells and is also readily eliminated from the blood circulation via glomerular filtration. This limits the access of interferon to Kupffer cells when it is systemically administered.

Kupffer cells express a number of mannose receptors on their surface. Interactions with these mannose receptors have been exploited in various drug delivery systems (DDS) (Higuchi et al., 2004). We previously produced a series of neoglyco-human serum albumins (HSA) to which one or three highly mannosylated oligosaccaride chains are attached (Man-HSA) by introducing the consensus sequence for the N-type oligosaccharide chains into the HSA gene using site-directed mutagenesis. These recombinant mutant HSAs proteins were subsequently successfully expressed in a *Pichia pastoris* yeast system (Hirata et al., 2010). Among them, a mutant that contains an Asp residue at position 494 was replaced by Asn (Man-HSA(D494N)) which contains highly mannosylated oligosaccharide chains. We anticipated that Man-HSA(D494N) might serve as a potent type-I interferon nanocarrier for Kupffer cell targeting because Man-HSA(D494N) was shown to be distributed efficiently in the liver, especially to Kuppfer cells, which can be attributed to the presence of highly mannosylated oligosaccharide chains, while such mannosylated chains would also cause a reduced glomerular filtration, derived from the association with HSA by albumination (Maruyama et al., 2016).

In this study, the N-terminal of interferon α2b (IFNα2b), an isoform of type-I interferon, was genetically fused to the C-terminal of Man-HSA(D494N) using albumin fusion technology, to create Man-HSA(D494N)-IFNα2b. This recombinant protein was then evaluated for its structural properties, pharmacokinetics (including Kupffer cell targeting ability), and anti-inflammatory and immunomodulatory activities derived from IFNα2b in the liver. Finally, the therapeutic efficacy of Man-HSA(D494N)-IFNα2b against Concanavalin A (Con-A) induced hepatitis model mice was evaluated.

## 2. Materials and methods

### 2.1. Materials

PfuTurbo DNA Polymerase was obtained from Agilent Technologies (Santa Clara, CA). The restriction enzymes of *BamH1* and *EcoR1* were purchased from Toyobo Co., Ltd. (Osaka, Japan). The restriction enzymes of *Ava1*, *Sal1* and *Xho1* and DNA Ligation Kit were purchased from Takara BIO Inc. (Kyoto, Japan). QIAGEN Plasmid Kits were purchased from QIAGEN, Inc. (Hilden, Germany). INTRON^®^ A was obtained from Merck & Co., Inc. (Kenilworth, NJ, USA). Mannan was purchased from Nacalai Inc. (Kyoto, Japan). All other chemicals and reagents used were of the highest commercially available quallity, and all solutions were made using deionized and distilled water.

### 2.2. Animals

ICR mice (male, 5 weeks) and C57BL/6 mice (male, 8 weeks) were obtained from Japan SLC, Inc. (Shizuoka, Japan).

### 2.3. Cell culture

RAW264.7 cells were cultured in DMEM medium containing 10% FBS, streptomycin and penicillin and maintained under 37 °C and 5% CO_2_. The medium was changed at 3 day intervals. The cells were passaged with a cell scraper after reaching confluence.

### 2.4. DNA recombination of man-HSA(D494N)-IFNα2b fusion protein

The designed fusion protein was composed of HSA(D494N) linked to IFNα2b via a polypeptide linker (-(GGGGS)_2_-). As previously reported, PCR was performed with a *PfuTurbo* DNA polymerase (Ikuta et al., 2010). To isolate the DNA fragment of the base sequence cording for HSA, restriction enzyme *Xho1* and *Ava1* recognition regions were inserted into the 5′ terminal and the 3′ terminal, respectively. An IFNα2b gene cDNA was cloned by mRNA extraction and reverse transcription from human kidney cells. To isolate the DNA fragment of the base sequence coding for IFNα2b, restriction enzyme *Ava1* and *EcoR1* recognition regions were inserted into the 5′ terminal and the 3′ terminal, respectively. The pPIC9 was digested with *Xho1* and *EcoR1*, and the appropriate side of the pPIC9 fragment was extracted by agarose gel electrophoresis. The cDNA construct cording for HSA-IFNα2b was produced by ligating DNA fragments (pPIC9, HSA and IFNα2b) overnight at 16 °C. The mutated HSA with mannose added was performed using a Quick Change kit (Agilent, CA), with mutagenic primers: 5′-GCTCTGGAAGTCAATGAAACATACG-3′ and 5′-CGTATGTTTCATTGACTTCCAGAGC-3′ (D494N, sense and antisense).

The pPIC9-mutated Man-HSA(D494N)-IFNα2b and the pPIC9K plasmid were then digested with *BamH1* and *Sal1*, the appropriate side of each fragment was extracted by agarose gel electrophoresis. Finally, each fragment was ligated using a DNA ligation kit, and *Pichia pastoris* (SMD1168 strain) was transformed with *Sal1*-digested pPIC9K-Man-HSA(D494N)-IFNα2b by electroporation according to the manual.

### 2.5. Expression and purification of the fusion protein

Expression and purification of Man-HSA(D494N)-IFNα2b was performed by the method as previously established for fusion proteins (Ikuta et al., 2010).

### 2.6. Evaluation of physical properties of fusion protein

SDS–PAGE was carried out using a 15% polyacrylamide gel. The fusion proteins were observed by staining with Coomassie Brilliant Blue (Nacalai Inc., Kyoto, Japan) and Periodic acid-Schiff (PAS). Western blotting was carried out with primary antibodies of anti-human IFNα2b and anti-HSA. It was then further reacted with secondary antibodies of anti-mouse IgG (IFNα2b) and anti-goat IgG (HSA) conjugated to HRP. The specific protein bands were visualized using a chemiluminescence kit. ESI-TOFMS analysis for Man-HSA(D494N)-IFNα2b was performed as previously reported (Miyamura et al., 2016).

### 2.7. RT-PCR analysis

The RNA extraction and cDNA preparation were carried out using RNAiso Puls (Takara Bio Inc., Siga, Japan) and PrimeScript^®^RT master mix (Takara Bio Inc., Siga, Japan), respectively. The mRNA levels of IL-10, IL-1Ra, PD-L1, and GAPDH were measured by RT-PCR analysis using a CFX connect™ Real-Time PCR detection system. PCR amplifications were carried out using SYBER^®^ Premix Ex TaqII (Takara Bio Inc., Siga, Japan). The threshold cycle (Ct) values for each gene amplification were normalized by subtracting the Ct value calculated for GAPDH. The primers used in this study are listed in Table 1.

**Table 1.** Sequence of primers for quantitative real-time RT-PCR.

Target gene	Forward primer (5′→3′)	Reverse primer (5′→3′)
Mouse IL-10	GGACAACATACTGCTAACC GACTC	AAAATCACTCTTCACCTGCTCCAC
Mouse IL-1Ra	TCAGATCTGCACTCAATGCC	CTGGTGTTTGACCTGGGAGT
Mouse PD-L1	TCAGCTACGGTGGTGCGGACT	AGCTTCTGGATAACCCTCGGCCT
Mouse GAPDH	AACTTTGGCATTGTGGAAGG	ACACATTGGGGGTAGGAACA

### 2.8. Radiolabeling of proteins with ^125^I

Man-HSA(D494N)-IFNα2b and HSA were radiolabeled with ^125^I according to the procedures reported previously (Watanabe et al., 2001). To adjust the protein dose appropriately, radiolabeled Man-HSA(D494N)-IFNα2b and HSA were diluted with the corresponding nonlabeled proteins.

### 2.9. Pharmacokinetics of man-HSA(D494N)-IFNα2b and HSA in mice

Mice were administered the ^125^I labeled proteins (1 mg/kg, ∼10^5^ cpm/mouse) via the tail vein. Blood samples were collected from the inferior vena cava at 0.05, 0.25, 0.5, 1 and 2 h after the injection of the ^125^I labeled proteins. Free ^125^I and degraded proteins were removed from the plasma by centrifugation in 40% TCA and 1% bovine serum albumin. Mice were sacrificed at each of these time points and the radioactivity of each organ was measured using a gamma counter.

### 2.10. Distribution of man-HSA(D494N)-IFNα2b in mice liver, and detection of mannose receptor and IFN receptor in mouse and human liver

The mouse livers were removed 1 h after FITC-labeled Man-HSA(D494N)-IFNα2b injection. Liver sections were fixed in 4% paraformaldehyde. After that, these sections were solublized in 0.1% Tween-TBS, followed by blocking using Block Ace for 60 min. These sections were then reacted with the primary antibodies (anti-mouse CD68 and anti-mouse CD31, 1:200; anti-mouse CD206 and anti-human CD206, 1:50; anti-mouse IFNAR1 and anti-human IFNAR1, 1:50) at 4 °C overnight, and with the secondary antibody (1:200) at RT for 90 min.

### 2.11. Effect of man-HSA(D494N)-IFNα2b on lethalCon-A challenge

To produce a lethal Con-A treated mice, Con-A (50 mg/kg) was injected to mice via the tail vein. Man-HSA(D494N)-IFNα2b (600 nmol/kg) was injected just prior to the Con-A treatment.

### 2.12. Effect of man-HSA(D494N)-IFNα2b on Con-A challenge

A Con-A was intravenously administrated to the prepared hepatitis model as previously reported (Maeda et al., 2015). The mice were administered 600 nmol/kg of Man-HSA(D494N)-IFNα2b, Man-HSA(D494N) or INTRON^®^ A via the tail vein Before Con-A injection. The dosage of INTRON^® ^ A was determined by calculating a unit showing IL-10 inducing activity comparable to that of Man-HSA(D494N)-IFNα2b in RAW264.7 cells. Moreover, the mice were administered Man-HSA(D494N)-IFNα2b at 2 h after the Con-A injection. Hepatic injury parameters, alanine aminotransferase (ALT) and aspartate aminotransferase (AST) levels in plasma were measured by a transaminase CII (Wako Chem., Saitama, Japan).

### 2.13. Western blotting

Total protein was extracted using a RIPA buffer containing 10 mM Tris-HCl (pH 7.4), 150 mM NaCl, 1% protease inhibitor cocktail, 1% Nonidet P-40. The protein concentration of the supernatant (protein fraction) was determined by a BCA protein assay. The protein and 3 × sample buffer were mixed, and boiled for 3 min before loading onto an SDS 15% polyacrylamide gel. After electrophoresis, proteins were transferred onto PVDF membranes and then blocked by 5% skim milk for 1 h, and washed with 0.1% Tween-TBS 3 times. The membranes were then reacted overnight with the primary antibody (anti-mouse IL-10, 1:5000; anti-mouse IL-1Ra, 1:5000) at 4 °C. The membranes were then washed and reacted with the secondary antibody at RT for 90 min. Using LAS 4000 mini (GE healthcare UK Ltd, Buckinghamshire, England), the intensity of each band was measured.

### 2.14. Isolation of kupffer cells from mice

Kupffer cells were isolated as previously reported (Dobashi et al., 1999). Briefly, a 0.25% collagenase solution were added liver specimen and it was shaken for 20 min in at 37 °C. After mixing in a Percoll solution (GE Healthcare, Uppsala, Sweden), the liver samples were centrifuged for 20 min at 500 × g. To remove RBCs, pellets were resuspended in the RBC lysis solution after removing the supernatant. Isolated mononuclear cells were cultured on Corning™ Primaria™ Tissue Culture Dishes (Corning, NY) to purify the Kupffer cells.

### 2.15. Measurement of the production of reactive oxygen species (ROS)

The production of ROS from Kupffer cells after the Con-A treatment was measured by flow cytometry. Isolated Kupffer cells were incubated with 0.5% CMH_2_DCFDA in Dulbecco’s PBS (DPBS) at 37 °C for 30 min and washed in DPBS. The cells were then applied to flow cytometry according to the instruction manual. In this study, the purity of Kupffer cells was calculated using markers that are co-expressed by Kupffer cells (CD68 or F4/80) by flow cytometry. To confirm the validity of this experimental system, we estimated the double-positive cells of CD68 and F4/80 in RAW264.7 and J774.1 cells, a typical macropharge cell line. The content of double-positive cells was >95% in both types of cells (Supplementary Figure S1).

## 3. Results

### 3.1. Design of man-HSA(D494N)-IFNα2b

**Figure 1.**
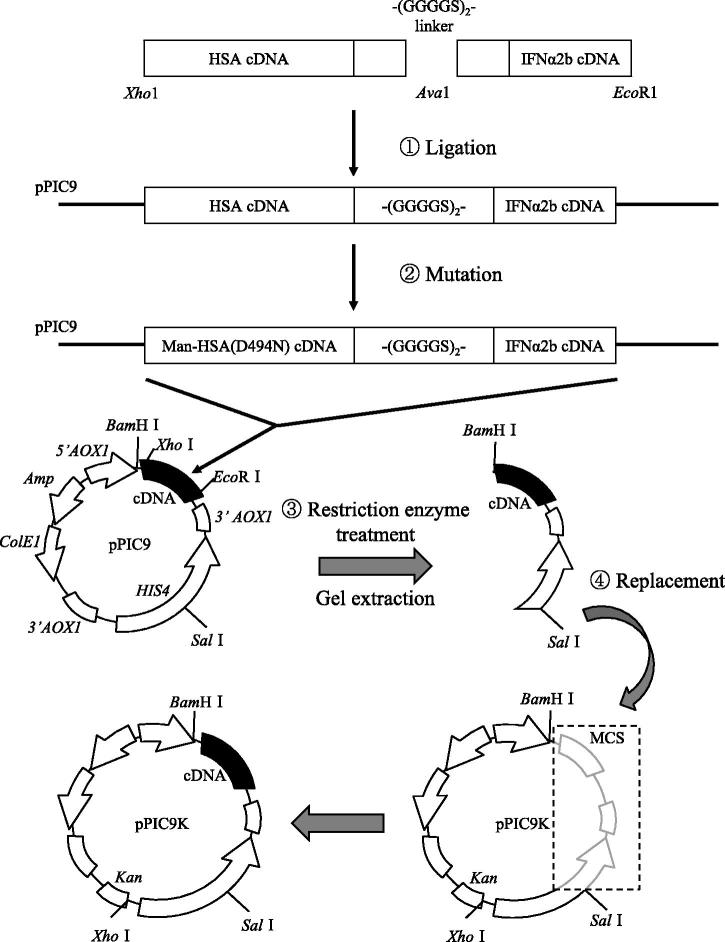
Flow chart describing the creation of the Man-HSA(D494N)-IFNα2b gene using the pPIC9K. MCS: multiple cloning sites

The Kupffer cell-targeting type-I interferon was designed and produced using a combination of two DNA recombinant techniques, namely albumin fusion technology and site-directed mutagenesis. As shown in Figure 1, a recombinant plasmid comprised of coding for DNA of HSA and IFNα2b was prepared by the ligation of the C-terminal of HSA-DNA fragments cut with *Xho1* and *Ava1* and the N-terminal of IFNα2b-DNA fragments cut with *Ava1* and *EcoR1* via the linker (Gly–Gly–Gly–Gly–Ser)_2_. It was then joined to pPIC9. Using the site-directed mutagenesis technique, the Asp unit at position of 494 in HSA was replaced with Asn to introduce the consensus sequence for N-linked oligosaccharide chains (hereafter referred to as ‘pPIC9-mutated Man-HSA(D494N)-IFNα2b’). To obtain the DNA fragment of the mutated Man-HSA(D494N)-IFNα2b, the pPIC9-mutated Man-HSA(D494N)-IFNα2b was digested with both *BamH1* and *Sal1*, then joined to pPIC9K (hereafter referred to as ‘pPIC9K-mutated Man-HSA(D494N)-IFNα2b’). The pPIC9K-mutated Man-HSA(D494N)-IFNα2b was then transformed into *Pichia pastoris* (SMD1168 strain) and the mannosylated recombinant fusion protein was produced using this expression system.

### 3.2. Structural properties of man-HSA(D494N)-IFNα2b

**Figure 2.**
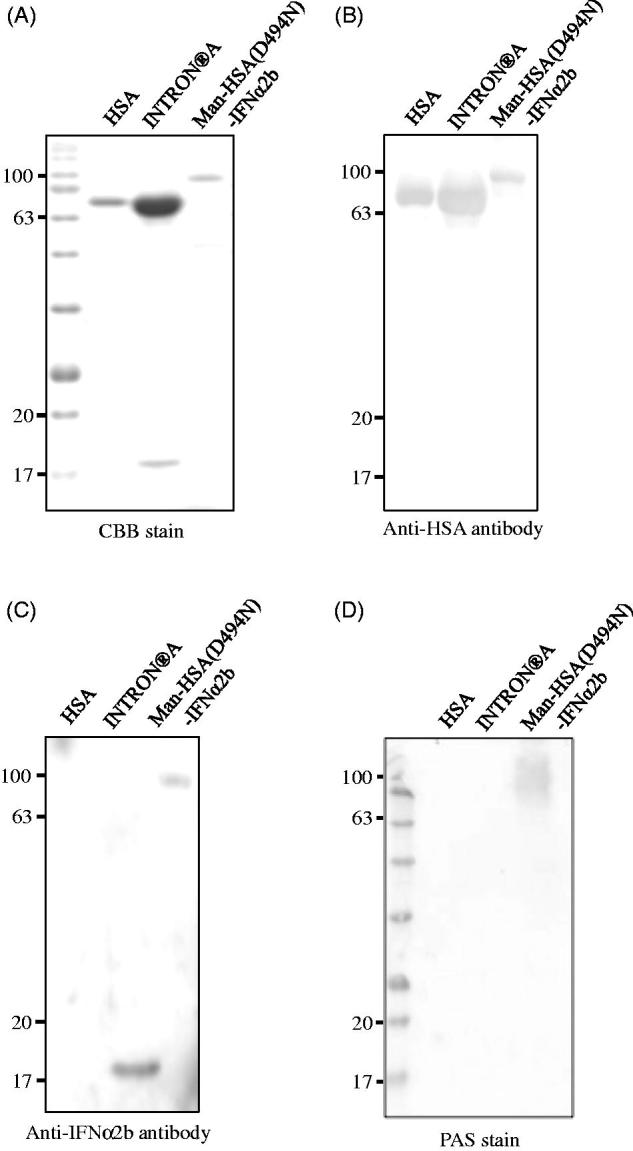
Structural properties of Man-HSA(D494N)-IFNα2b. (A,D) Human serum albumin (HSA), INTRON^®^ A and Man-HSA(D494N)-IFNα2b were electrophoresed, and the gels were also subjected to staining with (A) Coomassie Brilliant Blue (CBB) or (D) Periodic Acid-Schiff (PAS). (B,C) After being transferred to PVDF membranes, the samples were incubated with primary antibodies against (B) HSA or (C) IFNα2b. The lane of HSA and INTRON^®^ A were used as a positive control of fusioned HSA and IFNα2b.

The recombinant Man-HSA(D494N)-IFNα2b produced in this study was analyzed by CBB staining using HSA and a commercially available IFNα2b preparation (INTRON^®^ A: containing HSA as a pharmaceutical additive) as a control. CBB staining clearly showed that the position of the recombinant fusion protein band was higher than that of HSA (Figure 2(A)). To confirm the presence of HSA and IFNα2b in the fusion protein, Western blotting analyses were carried out using their antibodies. As shown in Figure 2(B,C), the anti-HSA antibody reacted positively with Man-HSA(D494N)-IFNα2b, HSA and INTRON^®^ A (upper band), while the anti-IFNα2b antibody reacted positively with Man-HSA(D494N)-IFNα2b and INTRON^®^ A (lower band). These results confirm that the fusion protein contained both HSA and IFNα2b.

To further confirm the presence of oligosaccharide chains in Man-HSA(D494N)-IFNα2b, Periodic acid-Schiff (PAS) staining was carried out. As shown in Figure 2(D), Man-HSA(D494N)-IFNα2b reacted positively with the PAS stain, while HSA did not. This indicates that oligosaccharide chains are present on the Man-HSA(D494N)-IFNα2b. To estimate the number of mannose residues in this oligosaccharide chains, ESI-TOFMS analysis was performed. ESI-TOFMS data showed that the molecular weight of the Man-HSA(D494N)-IFNα2b is 88573 Da that was higher than the theoretical molecular weight (86334 Da) of HSA-IFNα2b. In general, N-linked oligosaccharide chains added to *Pichia pastoris* are composed of 2 molecules of GlcNAc (MW:221 Da) and 8–14 molecules of Mannose (MW:180 Da) (Blanchard et al., 2007), therefore we estimated that Man-HSA(D494N)-IFNα2b is composed of 2 molecules of GlcNAc and approximately 10 molecules of Mannose.

### 3.3. Man-HSA(D494N)-IFNα2b retains type-I interferon activity *in vitro*

**Figure 3.**
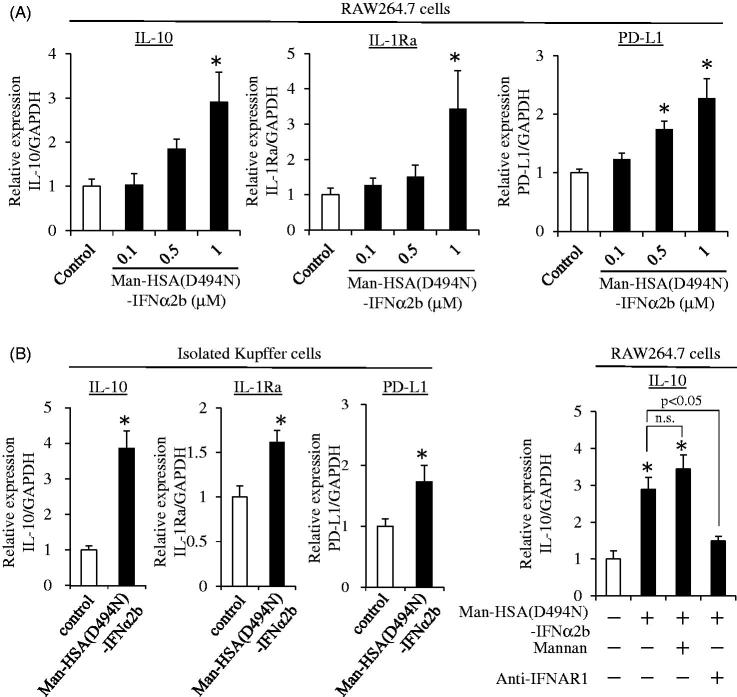
Man-HSA(D494N)-IFNα2b induce the production of mRNA of IL-10, IL-1Ra and PD-L1 on RAW264.7 and isolated Kupffer cells. IL-10, IL-1Ra and PD-L1 mRNA induction at 3 h after adding Man-HSA(D494N)-IFNα2b were evaluated on (A) RAW264.7 cells (*n* = 6) and (B) isolated Kupffer cells (*n* = 4). (C) IL-10 mRNA induction under the inhibition of the mannose receptor with Mannan (1 mg/mL) or the IFN receptor with anti-IFNAR1 antibody (20 μg/mL) on RAW264.7 cells (*n* = 6). *p < .05 as compared with control. n.s., no significant change.

When type-I interferon interacts with a macrophage through its receptor, IL-10, IL-1Ra and PD-L1 production is induced (Guarda et al., 2011; Petrasek et al., 2011; Ivashkiv & Donlin, 2014; Roh et al., 2014). Thus, to investigate whether the Man-HSA(D494N)-IFNα2b still retained the biological activities of IFNα2b, the mRNA levels of IL-10, IL-1Ra and PD-L1 were monitored by quantitative RT-PCR after adding Man-HSA(D494N)-IFNα2b to either RAW264.7 cells or isolated Kupffer cells isolated from mouse livers. As shown in Figure 3(A), at 3 h after the addition of Man-HSA(D494N)-IFNα2b (0.1 ∼ 1 μM), these mRNA levels increased in RAW264.7 cells and the increase was dose-dependent. Parallel results were also obtained from the isolated Kupffer cell (Figure 3(B)). These data revealed that the anti-inflammatory and immunomodulatory actions of type-I interferon were retained in the Man-HSA(D494N)-IFNα2b.

Next, to clarify whether the induction of IL-10 mRNA by Man-HSA(D494N)-IFNα2b resulted from its interaction with interferon receptors on the surface of macrophages or after being taken up by cells via the mannose receptor, we also investigated the effects of Man-HSA(D494N)-IFNα2b (1 μM) on mRNA induction in IL-10 in RAW264.7 cells in the presence of an excess amount of Mannan (1 mg/mL), which functions as an inhibitor of the mannose receptor, and as an interferon receptor neutralizing antibody (20 μg/ml). As shown in Figure 3(C), IL-10 mRNA levels were still increased by the Man-HSA(D494N)-IFNα2b, even in the presence of an excess amount of Mannan. In contrast, these inductions were abolished by pretreatment with the interferon receptor neutralizing antibody. This strongly suggests that Man-HSA(D494N)-IFNα2b induced the IL-10 production through the interferon receptor, and not after the internalization via the mannose receptor.

### 3.4. Man-HSA(D494N)-IFNα2b is preferentially distributed to kupffer cells

**Figure 4.**
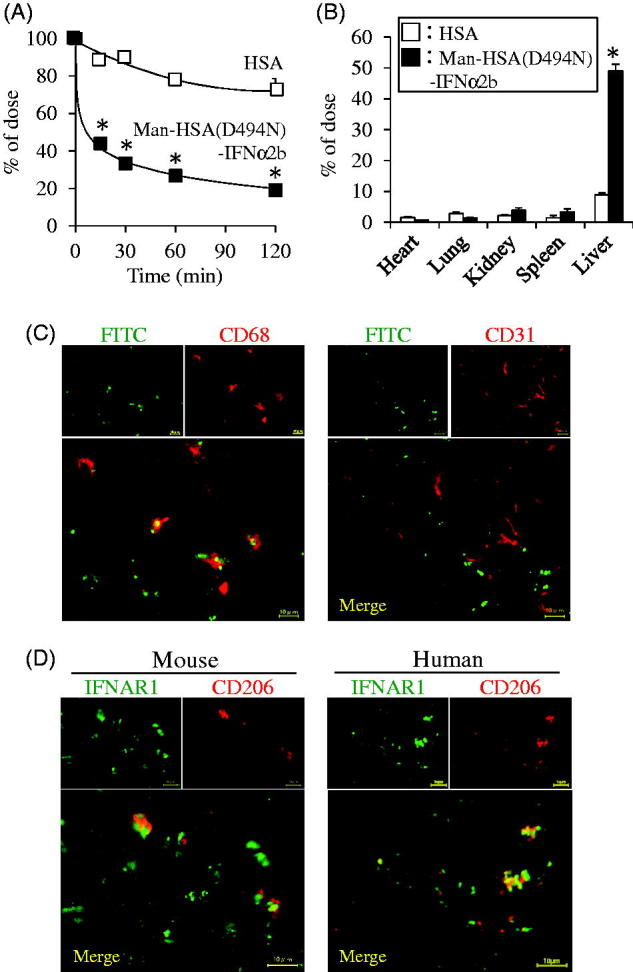
Man-HSA(D494N)-IFNα2b is distributed to the liver, and localized with Kupffer cells. Radioactivity of (A) plasma or (B) each tissue after the administration of ^125^I-labeled proteins to mice. The result of each tissue is at 15 min after administration. □: HSA, ▪: Man-HSA(D494N)-IFNα2b. Data are expressed the mean ± SEM (*n* = 4). *p < .05 compared with HSA. (C) The distribution of Man-HSA(D494N)-IFNα2b to CD68^+^ Kupffer cells or CD31^+^ vascular endothelial cells was analyzed by means of a fluorescence image technique using FITC-labeled Man-HSA(D494N)-IFNα2b (green) and anti-CD68 antibody (red) or anti-CD31 antibody (red). The areas of co-localization for each fluorescence are shown in yellow. (D) Co-localization of IFN receptor and mannose receptor in mouse or human liver was analyzed by fluorescence image technique using anti-IFNAR1 antibody (green) and anti-CD206 (mannose receptor) antibody (red). Magnification of liver sections were 180 times. Scale bar = 10 μm.

To characterize the pharmacokinetic properties of the Man-HSA(D494N)-IFNα2b, the ^125^I-labeled fusion protein was injected into the tail vein, and the radioactivity in plasma and tissues (heart, lung, liver, kidney, spleen) were determined. In this experiment, HSA was used as a control. As shown in Figure 4(A), compared to HSA, Man-HSA(D494N)-IFNα2b was more rapidly cleared from the blood circulation. At 15 min after the administration, the tissue distribution of the Man-HSA(D494N)-IFNα2b was examined (Figure 4(B)). Clearly, most of the radioactivity was found in the liver, approximately 5 times higher than that of HSA. A small portion of the radioactivity was also detected in the kidney and spleen.

To identify the localization of Man-HSA(D494N)-IFNα2b within the liver, FITC-labeled Man-HSA(D494N)-IFNα2b (600 nmol/kg) was intravenously administered to the mice. At 1 h after the injection, liver sections were prepared, and then, these were analyzed by fluorescence imaging technique. As shown in Figure 4(C) (upper-left panel), a substantial level of FITC fluorescence was observed. Since mannose receptors are mainly present on Kupffer cells and endothelial cells (Kogelberg et al., 2007), it is highly possible that the Man-HSA(D494N)-IFNα2b was distributed to these cells via mannose receptors. Thus, the sections of liver tissue were subjected to immunostaining with the anti-CD68 antibody, a marker of Kupffer cells (Krenkel & Tacke, 2017), and with the anti-CD31 antibody, a marker of endothelial cells. As shown in Figure 4(C), a part of the FITC fluorescence (green) was overlapped with the anti-CD68 antibody fluorescence (red) and appeared as yellow spots, whereas there was insignificant overlapping (yellow) between the FITC (green) and the anti-CD31 antibody (red). This suggests that Man-HSA(D494N)-IFNα2b was preferentially distributed in Kupffer cells than in endothelial cells.

Similar experiments using the anti-CD206 antibody, a marker of mannose receptor and the anti-interferon receptor antibody in mouse and human liver were conducted. As shown in Figure 4(D), the fluorescence derived from both markers was overlapped and appeared as yellow spots. This suggests that the Man-HSA(D494N)-IFNα2b possibly interacts with both mannose and interferon receptors on Kupffer cells.

### 3.5. Man-HSA(D494N)-IFNα2b effectively exerts hepato-protective actions in Con-A induced hepatitis mice

**Figure 5.**
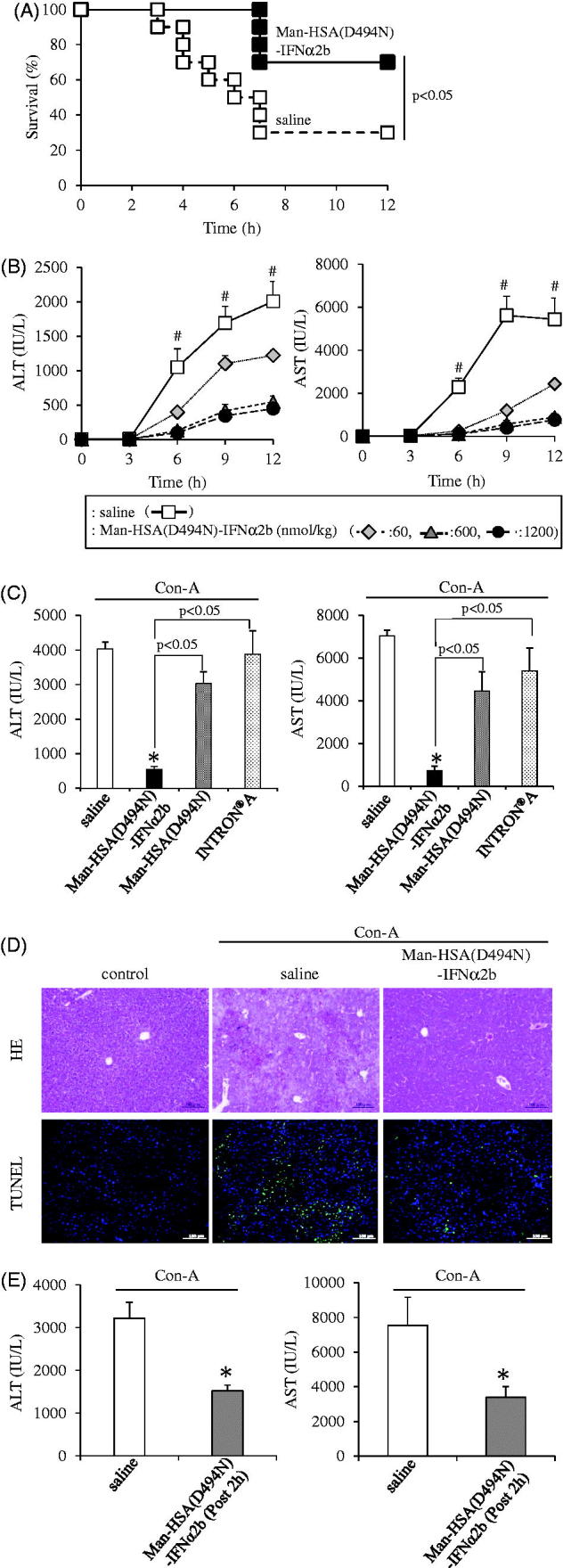
Man-HSA(D494N)-IFNα2b ameliorated Con-A induced liver damage in mice. (A) Man-HSA(D494N)-IFNα2b (600 nmol/kg) was administered intravenously just before lethal Con-A (50 mg/kg, *i.v.*) treatment. ▪: Saline, □: Man-HSA(D494N)-IFNα2b. Survival analysis was conducted according to Kaplan–Meier method and the log-rank test was performed on the differences between Saline and Man-HSA(D494N)-IFNα2b. Data are expressed the mean ± SEM (*n* = 10). (B–D) Man-HSA(D494N)-IFNα2b was injected intravenously just before the Con-A (12.5 mg/kg, *i.v.*) treatment. (B) ALT and AST values in plasma were measured at 0, 3, 6, 9 and 12 h after the Con-A challenge. (C) ALT and AST values in plasma were measured at 12 h after the Con-A treatment. (D) Paraffin-embed liver tissue were prepared at 12 h after the Con-A treatment and subjected to histopathological examination (HE and TUNEL staining). (E) Man-HSA(D494N)-IFNα2b was administered intravenously at 2 h after Con-A (12.5 mg/kg) treatment. Data are expressed the mean ± SEM (*n* = 6). #*p*<.05 compared with Man-HSA(D494N)-IFNα2b. *p < .05 compared with saline.

The hepato-protective action of Man-HSA(D494N)-IFNα2b was investigated using Con-A induced hepatitis model mice, a model for autoimmune, viral infection and drug-induced hepatitis (Nakashima et al., 2008). The mice were intravenously administered the Man-HSA(D494N)-IFNα2b (600 nmol/kg) just before administration of a lethal concentration of Con-A (50 mg/kg, *i.v.*). Figure 5(A) showed the survival rate of lethal Con-A challenged mice with the administration of saline or Man-HSA(D494N)-IFNα2b (600 nmol/kg). Obviously, the saline administered mice started dying at 3 h after the Con-A injection, and 70% of the mice had died at 12 h. In contrast, co-administration of Man-HSA(D494N)-IFNα2b resulted in significant increase in survival rate (70%).

To further investigate the mechanism responsible for the improved survival rate caused by the Man-HSA(D494N)-IFNα2b, the ALT and AST values in plasma were measured at 12 h after the low dose of Con-A (12.5 mg/kg, *i.v.*) treatment, because these values reached maximum at 12 h after the Con-A challenge. The Man-HSA(D494N)-IFNα2b (600 nmol/kg, *i.v.*) significantly decreased the ALT and AST values in plasma induced by Con-A and this inhibition was dose dependent (Figure 5(B)).

To clarify the contribution of each functional component of Man-HSA(D494N)-IFNα2b, similar experiments were performed using Man-HSA(D494N) or INTRON^®^ A (mixture of IFNα2b and HSA). As shown in Figure 5(C), neither the Man-HSA(D494N) nor the INTRON^®^ A significantly suppressed the elevation of the plasma ALT and AST on Con-A challenge. These results indicate that highly mannosylated oligosaccharide chains and IFNα2b are both required for the superior hepato-protective action of Man-HSA(D494N)-IFNα2b to be exerted.

Sections of liver tissue from Con-A model mice were histologically examined using HE and TUNEL staining (Figure 5(D)). As observed in the HE staining, the widely-spread necrotic region within the liver lobules were existed in the group treated with saline. However, the co-administration of Man-HSA(D494N)-IFNα2b largely suppressed these histological changes. Similar experiments using TUNEL staining revealed that TUNEL-positive hepatocytes were observed for the saline treatment group, whereas the numbers of the positive cells were decreased in the group that had been given the Man-HSA(D494N)-IFNα2b. These data suggest that Man-HSA(D494N)-IFNα2b prevents hepatic injury in Con-A-induced hepatitis mice.

Moreover, we investigated the effect of the postadministration of Man-HSA(D494N)-IFNα2b on the Con-A induced hepatitis model mice. Administration of Man-HSA(D494N)-IFNα2b (600 nmol/kg) at 2 h after the Con-A challenge (12.5 mg/kg, *i.v.*) significantly inhibited the increase in plasma ALT and AST values (Figure 5(E)). This indicated that even though the postadministration, Man-HSA(D494N)-IFNα2b (600 nmol/kg) exhibit the superior hepato-protective action.

### 3.6. Elucidation of the mechanism of hepato-protective action of Man-HSA(D494N)-IFNα2b

We next attempted to clear the mechanism responsible for the hepato-protective action of Man-HSA(D494N)-IFNα2b in Con-A induced hepatitis mice. As shown in Figure 5(B), the ALT levels were markedly increased at 3 h after the Con-A treatment, whereas the elevation of plasma ALT levels was significantly suppressed by the co- or postadministration of Man-HSA(D494N)-IFNα2b (600 nmol/kg, *i.v.*) (Figure 5(C,E)).

**Figure 6.**
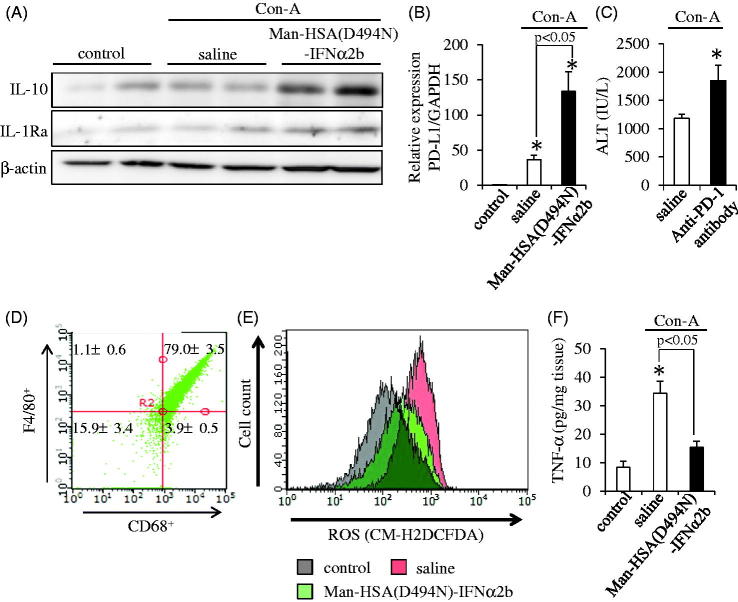
Man-HSA(D494N)-IFNα2b protects against Con-A induced liver injury by suppressed Kupffer cell activation. (A) Hepatic IL-10 and IL-1Ra were determined by Western blotting. (B) Hepatic PD-L1 mRNA levels were determined by real-time RT-PCR (*n* = 6). *p < .05 as compared with control. (C) Plasma ALT level was determined at 12 h after the Con-A challenge (*n* = 6). *p < .05 as compared with saline. (D) CD68 and F4/80 expression on isolated hepatic mononuclear cells were evaluated using flow cytometry. (E) ROS content within the Kupffer cells was determined by flow cytometry. (F) Protein expression of TNF-α in the liver was determined by ELISA (*n* = 6). *p < .05 as compared with control.

Therefore, the protein expression of IL-10 and IL-1Ra in liver at 3 h after the Con-A challenge was evaluated by Western blotting. As with the data from *in vitro* experiments using RAW264.7 and isolated mouse Kupffer cells, hepatic IL-10 and IL-1Ra levels were clearly increased as the result of the co-administration of Man-HSA(D494N)-IFNα2b (600 nmol/kg, *i.v.*) (Figure 6(A)).

In addition, the mRNA levels of PD-L1 in the liver were measured by quantitative RT-PCR at 3 h after the Con-A challenge. As shown in Figure 6(B), PD-L1 mRNA levels were also increased by the Man-HSA(D494N)-IFNα2b treatment. To confirm the effect of PD-L1 on Con-A induced liver injury, an anti-PD-1 antibody (5 mg/kg, *i.v.*) was administered to mice just prior to the injection of Con-A. As shown in Figure 6(C), the anti-PD-1 antibody caused a significant enhancement in plasma ALT values, suggesting that PD-L1 exerts a hepato-protective effect under these conditions.

Inflammatory cytokines, such as ROS and TNF-α, contribute to the development of hepatitis induced by Con-A. Thus, we also investigated the effect of Man-HSA(D494N)-IFNα2b on the elevated production of ROS in isolated Kupffer cells at 3 h after the Con-A treatment. In this study, the purity of Kupffer cells in the isolated mononuclear cells was calculated to be approximately 80% by the markers of Kupffer cells, CD68 or F4/80 (Figure 6(D)). As shown in Figure 6(E,F), Man-HSA(D494N)-IFNα2b effectively inhibited the elevated production of ROS levels by reducing TNF-α production.

## 4. Discussion

Type-I interferon has been widely used as an anti-viral agent for the treatment of hepatitis B and C. However, this drug has also been demonstrated to possess multifaceted anti-inflammatory and immunomodulatory properties that contribute to suppressing organ damage (Conrad et al., 2014; Ivashkiv & Donlin, 2014). In this study, we report on the development of a novel Kupffer cell-targeting type-I interferon, a Man-HSA(D494N)-IFNα2b fusion protein, that has enhanced hepatic targeting properties without compromising the anti-inflammatory and immunomodulatory effects of type-I interferon derived via the induction of IL-10, IL-1Ra and PD-L1.

When designing this Kupffer cell targeting type-I interferon, it is necessary to solve two obstacles, including the elimination via glomerular filtration and the recognition by Kupffer cells. These two issues were resolved in the present study by combining albumin fusion technology to increase the molecular weight of the protein and using site-directed mutagenesis to introduce mannose units into the protein to create a targeting effect. This was achieved by using a *Pichia pastoris* expression system to produce Man-HSA(D494N)-IFNα2b. It is well established that increasing the molecular size of a protein such as fusion with albumin can effectively reduce the rate of glomerular filtration of proteins or peptides with poor blood retention, including type-I interferon (Maruyama et al., 2016). In addition, the commercial IFNα2b preparation, for example, INTRON^®^ A, contains a large amount of HSA as a stabilizer, it is expected that IFNα2b would also be stable in the form of an albumin fusion molecule that contains recombinant Man-HSA(D494N)-IFNα2b.

In addition, once the cDNA of Man-HSA(D494N)-IFNα2b is established, the preparation procedures are the same as those for preparing recombinant HSA. Thus, Man-HSA(D494N)-IFNα2b is relatively easily prepared in a homogeneous state and good yield. Our results revealed that, after intravenous administration, the Man-HSA(D494N)-IFNα2b is predominantly and rapidly distributed to the liver, particularly to Kupffer cells (Figure 4(B,C)). This suggests that oligosaccharide chains are certainly attached to the fusion protein molecule. Subsequent examinations clearly showed that Man-HSA(D494N)-IFNα2b possesses highly mannosylated oligosaccharide chains (Figure 2(D)), similar to Man-HSA(D494N) and other recombinant glycoproteins that are expressed using *Pichia pastoris* (Hirata et al., 2010). This indicates that type-I interferon fusion did not interfere with the mannosylation of the recombinant HSA mutant. Consequently, the Man-HSA(D494N)-IFNα2b improves hepatic availability and hence should be able to dramatically enhance the pharmacological effects of IFNα2b in the liver.

IL-1Ra and its derivatives have attracted attention as novel anti-inflammatory agents (Petrasek et al., 2012; Lee et al., 2015) that inhibit inflammasomes that play an important role in a number of inflammatory diseases (Cui et al., 2015; Tilg et al., 2016). Recombinant IL-1Ra preparations have already received regulatory approvals for use in the treatment of rheumatoid arthritis, and various other drugs are presently in the clinical trial stages of evaluation (Lopalco et al., 2016). On the other hand, IL-10 and its inducers appear to be promising anti-inflammatory agents, and research regarding their practical application is currently underway (Saraiva & O’Garra, 2010; Mittal & Roche, 2015).

There are a number of drugs on the market that are known to be capable of inducing IL-1Ra or IL-10 expression. However, Man-HSA(D494N)-IFNα2b is superior to these products in terms of being able to induce the production of both IL-1Ra or IL-10 in the liver through the action of IFNα2b (Figures 3(A–C), 5(E) and 6(A)). Furthermore, these inductions occur rather quickly, reflecting the rapid and efficient hepatic distribution of Man-HSA(D494N)-IFNα2b (Figure 4(B)). It is noteworthy that Man-HSA(D494N)-IFNα2b exhibited superior therapeutic effects compared to IFNα2b against Con-A-induced hepatitis model mice (Figure 5(C)). In addition, the rescue effect of Man-HSA(D494N)-IFNα2b was observed in 2-h post-administration after Con-A injection (Figure 5(E)) and the lethal hepatopathy mice (Figure 5(A)). These promising results serve as proof-of-concept in developing a Kupffer cell-targeting type-I interferon for treating liver injury.

On the other hand, PD-L1 expression is induced by the action of type-I interferon on macrophages, which causes immune tolerance through interactions with PD-1 in T cells (Oikawa et al., 2007). The findings reported here show that Man-HSA(D494N)-IFNα2b also induced an increase in the mRNA level of PD-L1 in RAW264.7 cells, isolated Kupffer cells (Figure 3(A,B)) as well as in the livers of Con-A-induced hepatitis mice (Figure 6(B)). Therefore, it is possible that immune tolerance via the PD-1/PD-L1 pathway contributed to the suppressive effect of Man-HSA(D494N)-IFNα2b on liver damage. This is supported by the observation that anti-PD-1 antibodies were found to exacerbate hepatic injury in the Con-A-induced hepatitis model mice (Figure 6(C)).

Since mannose receptors play a critical role in the recognition of Man-HSA(D494N)-IFNα2b by Kupffer cells *in vivo*, it is reasonable to speculate that the effects of Man-HSA(D494N)-IFNα2b may be due to the initial binding between its mannose residues to mannose receptors and IFNα2b to adjacent interferon receptors on Kupffer cells. Studies using RAW264.7 cells revealed that Man-HSA(D494N)-IFNα2b continued to exert biological activities with respect to inducing mRNA of IL-10, even when excess Mannan was present as an inhibitor of mannose receptors. In contrast, such inductions were significantly suppressed in the presence of an interferon receptor inhibitor (Figure 3(C)). These observations indicate that mannose receptors are not involved in the induction of IL-10, IL-1Ra and PD-L1 by Man-HSA(D494N)-IFNα2b. During drug development, including DDS preparations, it is well recognized that drug candidates may not exert therapeutic effects in humans, even though the same compound had an excellent therapeutic impact in experimental animals. This discrepancy is due to species differences, where the structural and pathophysiological features of the drug target are different between the experimental animals and human. In this study, it is confirmed that the mannose (CD206) and interferon receptors, which are equivalently co-localized on Kupffer cells in both mouse and human livers, were the target molecules of Man-HSA(D494N)-IFNα2b.

It is also well known that mannose receptors (CD206) are not only expressed on Kupffer cells in the liver, but also on endothelial cells (Azad et al., 2014). The present results indicate that Man-HSA(D494N)-IFNα2b preferentially interacts with Kupffer cells (Figure 4(C)). This could be due to the different type of mannose receptors that are expressed in these two types of cells, and their own distinctive adaptability to oligosaccharide chains of Man-HSA(D494N)-IFNα2b. Dectin-2, mannose receptor specifically expressed on Kupffer cells or Dendritic cells, have a sugar recognition domain which is localized at the inner part of the folded from of their extracellular structure (McGreal et al., 2006). Because of such a unique structural feature, Dectin-2 receptors have a high binding affinity for ligands with clusters of mannose units that contain more than seven residues, and its specificity is enhanced with increasing number of mannose residues. As oligosaccharide chains of Man-HSA(D494N)-IFNα2b have a mannose cluster, it would be expected that Man-HSA(D494N)-IFNα2b is preferentially recognized by cooperating with CD206 and Dectin-2 on Kupffer cells.

Macrophages are classified into M1- and M2-polarized subtypes. M1-type macrophages have inflammatory functions, whereas M2-type macrophages have anti-inflammatory functions. Since CD206 is a marker for M2-type macrophages, it would be interesting to know whether Man-HSA(D494N)-IFNα2b exerts any influence on the distribution of this population of hepatic macrophage subtypes. As shown in Figure 6(A), after administering Man-HSA(D494N)-IFNα2b to Con-A-treated mice, the production of IL-10 markedly increased. Since IL-10 is also a marker of M2-type macrophages, the question arises as to whether such an elevation in IL-10 caused by Man-HSA(D494N)-IFNα2b might also cause an increase in the population of M2-type macrophages. If this is correct, the repeated administration of Man-HSA(D494N)-IFNα2b would facilitate its hepatic distribution by shifting the macrophage population in the direction of the M2-phenotype of hepatic macrophages. Thus, we plan to clarify this issue in the future study using chronic hepatitis models.

The recent development of anti-viral hepatitis agents has been remarkable. The focus of hepatitis treatment development in the future is expected to shift to nonviral forms of hepatitis such as alcoholic liver disease (Louvet & Mathurin, 2015), nonalcoholic steatohepatitis (Oseini & Sanyal, 2017) and autoimmune hepatitis, in which there are presently no established therapeutics available. Since IL-10, IL-1Ra and PD-L1 have been recognized as promising potential treatments for these conditions, Man-HSA(D494N)-IFNα2b would be expected to be effective against the various forms of chronic liver diseases.

## 5. Conclusions

This study is the first report of a successful attempt to produce a novel Kupffer cell targeting type-I interferon, which is comprised of a fusion protein of type-I interferon and HSA with a highly mannosylated N-linked oligosaccharide chains, using combined albumin fusion technology, techniques of site-directed mutagenesis and an expression system using *Pichia pastoris*. The resulting Man-HSA(D494N)-IFNα2b exhibited avoidance of the glomerular filtration and improved specific interaction with Kupffer cells and consequently exerted superior hepato-protective effects, by virtue of the multifaceted anti-inflammatory and immunomodulatory actions of type-I interferon. It therefore appears that Man-HSA(D494N)-IFNα2b has considerable potential for use as an effective treatment of chronic liver diseases.
